# Quantitative assessment of global and regional strain in relation to infarct size in patients with myocardial infarction

**DOI:** 10.1186/1532-429X-13-S1-P131

**Published:** 2011-02-02

**Authors:** Nabil A Shafi, Kathleen Bertman, Andrew Yoon, Rena Toole, Matthew Ronin, Simcha Pollack, Nathaniel Reichek, Madhavi Kadiyala

**Affiliations:** 1 St. Francis Hospital- The Heart Center, Roslyn, NY, USA; 2 St. Francis Hospital- The Heart Center, Roslyn, NY and, St. John's University, New York City, NY, USA; 3 St. Francis Hospital- The Heart Center, Roslyn, NY and, Stony Brook University School of Medicine, NY, USA

## Introduction

Assessment of regional and global left ventricular (LV) function and strain are important in the setting of prior myocardial infarction (MI). Feature tracking (FT-MRI) is a novel cardiac MRI method for assessment of myocardial strain, similar to speckle tracking in 2-D echocardiography, which provides multi-planar strain data without the need for tagged images.

## Purpose

The goal of our study was to characterize LV global and regional strains in patients with prior MI using FT-MRI and determine the relationship of strains to infarct size, location and LV ejection fraction (EF).

## Methods

Eighty patients with history of past MI were grouped by MI coronary territory {left anterior descending (LAD), left circumflex (LCX), right coronary (RCA)}. Scar quantification was done by computer-assisted planimetry (Medis Qmass v7.2) of gadolinium enhanced delayed images and MI percentage was determined. Global and regional circumferential subendocardial (Ecc Endo) and subepicardial (Ecc Epi) and longitudinal (Ell) strains were derived from three long axis (4, 3 and 2 chamber) and three short axis (basal, mid, apical) steady state free procession cine planes. Semi-automated tracing of endocardial and epicardial borders using FT-MRI (Diogenes MRI, Tomtec Systems) was performed and strains were mapped to a 17 segment AHA model. Repeated measures ANOVA was used to compare normal strain values from 60 healthy subjects to MI patients controlling for scar percentage.

## Results

The mean age was 64.4(12) years and mean EF was 42.1%(11). Peak global Ecc Epi and Ecc Endo were decreased in all MI patients, except in Ecc endo LCx infarcts, compared to normals (all with p≤ 0.01) (Table 1). Peak Ell strain was relatively preserved in the majority of infarcts except in LAD infarcts, where it was significantly decreased in the LAD territory (p =0.01). The best predictor of reduced LVEF in MI patients was reduced global Ecc Endo (r =0.82, p<0.0001), followed by global Ecc Epi (r=0.64, p <0.0001), scar territory percent (r=0.53, p<0.0001) and global Ell (r=0.43, p< 0.0001).

**Table 1:** 

**Patient Demographics and Peak Strain Values**	**Normals** (N=60)	**LAD Infarct** (N=22)	**LCx Infarct** (N=11)	**RCA Infarct** (N=31)	**Multi infarct** (N=16)
**Age** (years)	54.4 (14)	61.9 (10)	63.2 (9)	62.7 (15)	70.0 (7)
**LVEF** (%)	58.2 (5)	38 (8)	50.1 (10)	43.9 (12)	36 (11)
**Scar** (%)	NA	23.1 (11)	10.2 ± (6)	14 (7)	19 ± (8)
**Ell** Avg (SD)					
Global	-16.2 (5)	-9.4 (3) **P=0.04**	-10.2 (5)	-14.7 (5)	-8.6 (4)
Basal	-14.0 (8)	-16.1 (5)	-8.1 (7)	-12 (6)	-8 (6)
Mid	-14.1 (8)	-10.6 (3)	-6.7 (5)	-11.4 (6)	-7.1 (5)
Apical	-17.1 (8)	-5.7 (2) **P=0.01**	-13.0 (4)	-16.5 (6)	-9.2 (5)
LAD	-18.8 (6)	-8.8 (3) **P=0.01**	-14.2 (5)	-17.6 (5)	-9.7 (4)
LCx	-18.7 (8)	-10.0 (5)	-12.6 (6)	-19 (7)	-10 (5)
RCA	-12.2 (10)	-10.8 (5)	-5.1 (4)	-8.5 (7)	-6.6 (5)
**Ecc Epi**					
Global	-16.1 (3)	-8.4 (3) **P=0.001**	-10.6 (3) **P=0.01**	-9.6 (4) **P=0.001**	-7.8 (3) **P=0.026**
Basal	-14.8 (3)	-10.4 (3) **P=0.006**	-10.3 (3)	-8.2 (4) **P=0.006**	-7.7 (3) **P=0.004**
Mid	-15.6 (4)	-8.7 (3)	-8.1 (3) **P=0.002**	-7.8 (4) **P<0.001**	-7.1 (3) **P<0.001**
Apical	-19.3 (5)	-6.9 (4) P**<0.004**	-14.9 (5)	-15.8 (8) **P=0.002**	-9.5 (7)
LAD	-17.1 (5)	-6.6 (3) **P=0.007**	-13.2 (3)	-11.9 (6) **P=0.002**	-8.1 (4)
LCx	-22.5 (7)	-13.3 (6) **P=0.03**	-10.9 (£) **P=0.02**	-15.5 (7) **P=0.01**	-11.9 (6)
RCA	-10/4 (4)	-8.9 (3)	-7.7 (3)	-6.0 (2) **P=0.002**	-6.2 (2)
**Ecc Endo**					
Global	-24.0 (4)	-13.7 (5) **P=0.004**	-18.0 (4)	-16.1 (6) **P=0.001**	-11.8 (5) **P=0.03**
Basal	-23.8 (5)	-17.2 (5) **P=0.04**	-171. (4)	-13.8 (6) **P=0.007**	-12.2 (5) **P=0.05**
Mid	-23.0 (5)	-18.8 (5)	-15.2 (5)	-13.5 (6) **P=0.004**	-10.4 (4) **P=0.04**
Apical	-27.1 (7)	-11.3 (6) **P=0.014**	-24.5 (8)	-24.5 (10)	-14.4 (9)
LAD	-26.9 (6)	-10.7 (6) **P=0.02**	-22.1 (5)	-18.9 (8) **P=0.004**	-12.2 (5)
LCx	-25.0 (7)	-19.0 (6)	-17.3 (3)	-21.7 (8)	-14.3 (6)
RCA	-20.7 (4)	-15.6 (6)	-14.4 (4)	-11.8 (7)	-11.2 (5)

## Conclusion

FT-MRI permits detailed assessment of global and regional strains in patients with MI. Global Ecc was decreased in all MI patients, while Ell was preserved in the majority on infarcts except LAD infarcts. The best predictor of reduced LV EF was reduced global Ecc Endo strain, which was superior to infarct size.

